# Solid trouble: tau and TDP-43 interaction in aggregation and pathology

**DOI:** 10.1038/s44318-025-00646-3

**Published:** 2025-11-24

**Authors:** Nicolas L Fawzi

**Affiliations:** Department of Molecular Biology, Cell Biology & Biochemistry and Robert J. and Nancy D. Carney Institute for Brain Science, Brown University, Providence, RI USA

**Keywords:** Molecular Biology of Disease, Neuroscience

## Abstract

New research in *The EMBO Journal* provides insights into the interactions of tau and TDP-43 in neurodegenerative diseases.

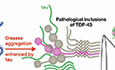

## Teaser

Both microtubule-binding protein tau and RNA-binding protein TDP-43 have been associated with neurodegenerative diseases, such as Alzheimer’s disease, including in co-pathological deposition with each other, but how they interact biophysically and pathologically has been challenging to evaluate. In this issue of *The EMBO Journal*, Simonetti and colleagues show how tau and TDP-43 directly interact both in phase separation and pathologically associated seeding, resulting in enhanced TDP-43 aggregation.

Continued advances in the treatment and prevention of infectious diseases and cancer combined with efforts in nutrition and public health across the globe have given a promise of longer life expectancy. Yet, these successes have prompted a focus on the growing incidence of neurodegenerative diseases in aging populations, where treatment options are currently few. Much of the challenge in developing therapies for neurodegenerative diseases stems from a difficulty in identifying their molecular causes—how these conditions are initiated, spread, and eventually result in neurodegeneration. Although Alzheimer’s disease (AD), the best known and most studied among these conditions, has been described and studied for more than 100 years, the fundamental features of its etiology remain challenging to probe, stymying therapeutic progress. A common feature of many of these diseases is the accumulation of proteins into neuronal inclusions. Interestingly, several conditions including AD show evidence for co-occurring protein pathologies. In their new study, Simonetti et al (2025) use biochemical and cellular assays to probe how co-depositing proteins may pathologically interact to direct co-assembly and aggregation, contributing to neurotoxicity.

Extracellular plaques composed of amyloid-β fibrils and intracellular tangles of tau are key hallmarks of AD—and their role in disease has been extensively debated (Busche and Hyman, 2020). Inclusions of the RNA-binding protein TDP-43 have been identified as the main hallmarks of amyotrophic lateral sclerosis (ALS) (Mackenzie et al, 2007), setting in motion a tide of studies showing that TDP-43 inclusions are also the primary features of additional conditions including subtypes of frontotemporal dementia and even traumatic brain injury. Evidence that familial forms of ALS can be caused by missense mutations in TDP-43 adds to the strong case that TDP-43 accumulation contributes to these diseases. Recently, neuropathologic change associated with limbic-predominant age-related TDP-43 encephalopathy (LATE-NC), including TDP-43 accumulation, has been linked to clinical symptoms typical of AD (Nelson et al, 2019). Importantly, tau and TDP-43 pathology also frequently co-occur in patient brains, but understanding of their interaction—molecular origins, physical contacts, and downstream pathways—has not yet emerged. What is clear from the observation of mixed pathologies is that models focusing only on a single disease protein (e.g., tau or TDP-43) may miss important cross-talk between these players that may occur before co-deposition. Indeed, researchers have long suggested that tau oligomers too small to easily detect may contribute to toxicity (Brunden et al, 2008), while rare TDP-43 variants may cause dysfunction without obvious cytoplasmic aggregation (Arnold et al, 2013). If dysfunction attributed to the single protein can occur without inclusion formation, an understanding of how tau and TDP-43 interact before inclusion formation as well as how they influence each other’s aggregation may provide insights into their potentially intertwined roles in disease.

**Figure 1:**
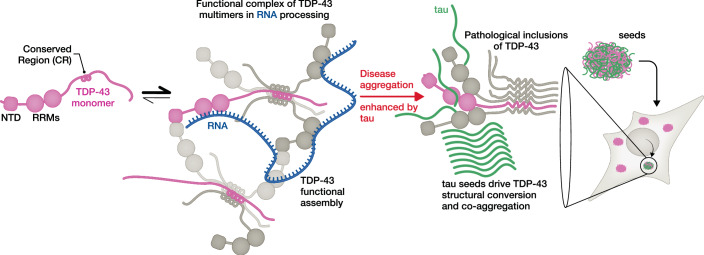
TDP-43 and tau co-assemble in condensates and co-aggregate in disease. TDP-43 and tau form dynamic condensates via multivalent interactions. In the case of TDP-43, these condensed forms are associated with RNA processing functions. While TDP-43 suppresses tau fibril propagation, tau fibril seeds drive TDP-43 to convert to aggregated forms in biochemical experiments and in cellular seeding assays. These insights may provide molecular details on TDP-43 and tau co-pathology in neurodegeneration.

Simonetti et al probe how direct interaction between TDP-43 and tau alters their phase separation and aggregation in vitro and in cells, putting forward evidence of a link between physical interactions and pathology (Fig. 1). Both TDP-43 and tau are capable of forming liquid-like biomolecular condensates (Wang et al, 2018; Wegmann et al, 2018). Simonetti et al (2025) show that, in biochemical assays, tau and TDP-43 each enhance the condensation of the other. Tau further enhances the conversion of TDP-43 into amorphous aggregates in biomolecular co-condensates. This effect is stimulated by phosphorylation of tau and proceeds via direct interactions. Conversely, TDP-43 appears to suppress tau fibrilization, suggesting that tau fibrils or seeds bind to TDP-43 in a competitive fashion. Truncated versions of tau and TDP-43 disrupt these processes, suggesting that multisite interactions between the domains of both proteins contribute to their interactions. The aggregation-prone C-terminal domain of TDP-43 that forms the core of the inclusions in TDP-43 diseases is not sufficient to alter tau aggregation, suggesting that functional TDP-43 may serve an important role in modulating tau propagation. Indeed, sequestration of TDP-43 in inclusions in cases with tau and TDP-43 co-pathology may lead to exacerbated tau seeding. Together, these biochemical studies map out the interactions between these proteins and their potential significance.

Importantly, the authors assess whether these molecular interactions play significant roles in established cellular models of pathological inclusion seeding. In correspondence with their finding that TDP-43 suppresses tau aggregation in test tubes, TDP-43 also inhibits tau seeding in cell lines expressing two forms of tau each tagged with a different fluorescent protein, providing spectroscopic evidence of pathological tau self-assembly on the nanometer scale. Importantly, extracts of brains with TDP-43 and tau co-pathology (e.g., from AD with LATE-NC) also show suppressed tau seeding compared to extracts from patient brains without TDP-43 pathology. Demonstrating specificity, tau seeding is not observed for extracts from Parkinson’s disease patients, suggesting that neurodegeneration alone is not sufficient to cause the observed seeding, which implies that brain samples from AD patients contain material capable of specifically seeding that is modulated by co-pathology. Paralleling their biochemical findings, the authors find that TDP-43 seeding is robustly stimulated by extracts with tau and TDP-43 co-pathology, similar to what is seen for extracts from frontotemporal dementia patient brains with TDP-43 inclusions only.

These findings suggest that the interactions between tau and TDP-43 probed in the recombinant proteins may also be found in cells, motivating further work to understand the atomic details of these interactions. Although the authors used truncation variants and cross-linking mass spectrometry, technical challenges like TDP-43’s lack of cross-linking residues and trypsin sites in the critical C-terminal domain prevent complete coverage of the detailed contacts. Future work combining additional specific variants of tau and TDP-43 (e.g., that disrupt TDP-43 RNA binding or prevent functional assembly of the N-terminal or C-terminal domain) with structural studies of dynamic and amorphous assemblies (e.g., Förster resonance energy transfer or nuclear magnetic resonance spectroscopy) should be able to shed more light on the mechanism, including on how tau perturbs the physiological assembly of TDP-43 (Rizuan et al, 2025).

By performing both biochemical and cellular seeding studies, this work also highlights the challenges in linking biophysical mechanisms to cellular outcomes, a major issue across studies of neurodegenerative diseases. These data provide more evidence for the view that phase separation and aggregation experiments looking at single proteins may miss important biological context (Alberti et al, 2025). Indeed, even adding one other partner alters the biochemical properties, emphasizing the complexity of interactions mediating condensation and aggregation, and opening questions about what additional players are needed to understand the dysfunction of the proteins forming inclusions in disease. Despite these challenges, with the molecular pathways initiating cellular toxicity remaining to be resolved, but with evidence that protein aggregation co-pathology is a key feature across distinct conditions, the experiments here provide a useful template for future studies.

These data, however, also draw attention to challenges in model systems. Prior studies have also suggested that cases of AD (e.g., exhibiting tau pathology) with co-occurring LATE-NC (i.e., TDP-43 accumulation) show increased tangle burden and tau phosphorylation (Tomé et al, 2023). That work and the new study (Simonetti et al, 2025) are both consistent with TDP-43 seeding from brains with TDP-43 and tau co-pathology. However, the former shows that extracts from tau/TDP-43 co-pathology enhance rather than inhibit tau seeding. Understanding whether this discrepancy arises from variability in patients, in sample handling (Simonetti and colleagues here worked to refine the extraction method and reduce variability in normalization across different patient conditions), or in specific brain regions tested requires additional studies, including with different models of tau seeding. Furthermore, more work is needed to understand the molecular origins of the seeding. Although the presence of TDP-43 material is confirmed in the insoluble preparations, its precise potency and nature remain to be characterized. Solving these challenges is a tall order that will require sustained efforts—the growing number of people suffering from these devastating conditions motivates further effort.
